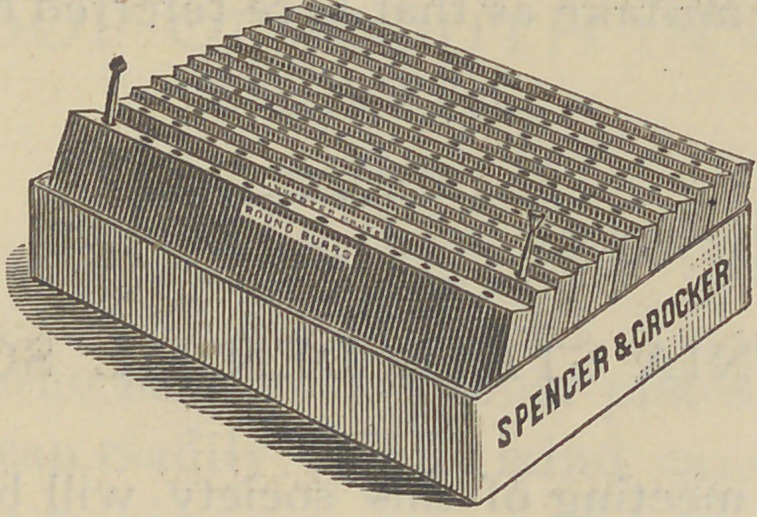# Something New

**Published:** 1878-12

**Authors:** 


### SOMETHING NEW.

Dr W. S. How, of this city, has devised and made a little appliance, to which I will give the name of engine-burr-holder, as this will give an idea of its use.

It consists of a rectangular box, three by four inches, and one inch deep, neatly made and plated.

In this are fitted twelve slides of wood, an inch and a half wide, and about two lines thick; in each of these there is twelve holes, to receive the burrs, drills, etc., thus making a convenient rack or holder for one hundred and forty-four instruments.

It is the neatest and most convenient arrangement for this purpose I have seen.

## Figures and Tables

**Figure f1:**